# Association between polymorphism rs11200638 in the HTRA1 gene and the response to anti-VEGF treatment of exudative AMD: a meta-analysis

**DOI:** 10.1186/s12886-017-0487-2

**Published:** 2017-06-21

**Authors:** Ya-li Zhou, Chun-li Chen, Yi-xiao Wang, Yao Tong, Xiao-ling Fang, Lin Li, Zhao-yang Wang

**Affiliations:** 1 Department of Ophthalmology, Shanghai Ninth People’s Hospital, Shanghai Jiaotong University School of Medicine, Shanghai, 200011 China; 2 Department of Ophthalmology, Xinhua Hospital, Shanghai Jiaotong University School of Medicine, Shanghai, China; 3 Department of Ophthalmology, Shengli Oilfield Central Hospital, Dongying, Shandong China; 4 Department of Ophthalmology, Shanghai Eye Hospital, Shanghai, China

**Keywords:** Anti-VEGF, Exudative AMD, HTRA1 gene, Treatment response

## Abstract

**Background:**

Anti-angiogenesis treatments are the most commonly used treatments for the vision loss caused by exudative age-related macular degeneration (AMD), in which the anti-vascular endothelial growth factor (VEGF) drugs with ranibizumab and bevacizumab are current standard treatments. However, the outcome of anti-VEGF therapeutics is not uniform in all patients.

**Methods:**

We performed a literature-based meta-analysis including, five published studies relevant to HTRA1 and response to anti-VEGF treatment (bevacizumab or ranibizumab). Summary odds ratios (ORs) and 95% confidence intervals (CIs) were estimated using fixed- and random-effects models. Sensitivity analysis and meta-regression were also performed. Q-statistic test and Egger’s test was used to evaluate heterogeneity and publication bias respectively.

**Results:**

Overall, no association between the rs11200638 polymorphism in HTRA1 gene and the anti-VEGF treatment response was found in the genotype GG versus AA (OR = 1.06; 95% CI: 0.77 to 1.48; *P* = 0.98), genotype GA versus AA (OR = 1.11; 95% CI: 0.83 to 1.47; *P* = 0.93), genotype GG + GA versus AA (OR = 1.22; 95% CI: 0.94 to 1.57; *P* = 0.09), and allele G versus A (OR = 0.92; 95% CI: 0.78 to 1.08; *P* = 0.14). In the subgroup analysis by ethnicity Caucasian population, and a significant association was still not observed in all genetic models. Sensitivity analysis indicated the robustness of our findings, and no publication bias was observed in our meta-analysis.

**Conclusions:**

This study shows that there was no association between the polymorphism rs11200638 in HTRA1 gene and response to anti-VEGF treatment of exudative AMD. However, more studies are needed to further prove the conclusion of present study, especially well-designed and high quality randomised controlled trials or intervention studies.

## Background

Age-related macular degeneration (AMD) is a leading cause of irreversible blindness in older individuals worldwide [1, 2]. AMD can be divided into early and advanced stages, according to the clinical features. There are two main categories of advanced AMD: atrophic AMD (dry AMD) and neovascular AMD (exudative AMD). Both of these conditions result in the loss of central vision, but the majority of severe vision loss occurs in exudative AMD, which is characterized by choroidal neovascularization (CNV) [3, 4]. The exact etiology of AMD is remained unknown despite the major risk factors for AMD including advanced age and smoking, with age being the strongest risk factor. In recent years, multiple studies have confirmed that genetic factors also play a substantial role in the etiology of AMD [5–7], such as single nucleotide polymorphisms (SNPs) in the complement factor H (CFH) [8, 9], age-related maculopathy susceptibility 2 (ARMS2) [10, 11], and high temperature requirement factor A1 (HTRA1)genes [12]. There is already strong evidence that the rs11200638 polymorphism, located in the HTRA1 promoter region [13], can increase susceptibility to AMD, especially the neovascular type, in Caucasian and Asian populations [14–16]. Possession of the high risk A allele of the HTRA1 gene is related with increased levels of the HTRA1 protein in drusen, the retinal pigment epithelium (RPE) and the choroidal neovascular membranes of eyes with AMD [12]. Therefore, it is possible that over-expression of HTRA1 could change the integrity of Bruch’s membrane and accelerate the development of CNV [17]. In addition, HTRA1 inhibits transforming growth factor-β, which regulates angiogenesis [17]. A large cohort of Caucasian patients, Zhang et al. suggested that the HTRA1 gene plays a critical role in angiogenesis through transforming growth differentiation factor 6 (GDF6) belonging to growth factor-βfamily member [18].

Anti-VEGF treatment with ranibizumab or bevacizumab are current standard treatments for the vision loss caused by CNV [19–21]. However, the outcome of anti-VEGF therapeutics is not uniform in all patients. Research were performed to investigate the associations of genetic variants with different response patterns to determine whether genetic variations could predict treatment response and therefore provide personalized therapy. Studies have shown that CFH, ARMS2, HTRA1, vascular endothelial growth factor A (VEGFA), vascular endothelial growth factor receptor 2 (VEGFR2) are all associated with anti-VEGF treatment outcomes [22–24], meanwhile a few literature-based meta-analyses indicated that CFH and ARMS2 polymorphisms might be associated with treatment response and outcomes in exudative AMD [25, 26].

To date, a few studies have also indicated that rs11200638 in the HTRA1 gene could influence patients’ responses to treatment with anti-VEGF drugs for exudative AMD. However, the treatment effect remains controversial. Some studies have identified significant associations between genetic variants of the rs11200638 polymorphism and patient response [23, 24, 27]. In addition, those studies suggested that the HTRA1 promoter SNP (rs11200638) were associated with a poorer visual outcome for ranibizumab or bevacizumab treatment in neovascular AMD, suggesting strong pharmacogenetic associations with anti-VEGF treatment. However, other study has not found any significant associations between HTRA1 and treatment outcomes among different genotypes [28]. As we know, no systematic reviews or meta-analyses have been published to evaluate the relationship between rs11200638 polymorphisms in the HTRA1 gene and the response to anti-angiogenesis treatment for exudative AMD. Here, we conducted a meta-analysis aiming to combine individual studies and to demonstrate the association more precisely.

## Methods

### Search strategy and inclusion criteria

This meta-analysis was conducted according to the Preferred Reporting Items for Systematic Reviews and Meta-Analyses (PRISMA) Guidelines [29]. We searched in PubMed, Web of Science, and Embase with no limitations on language. The following combinations of relevant key terms in the article were searched: (HTRA1 OR high temperature requirement factor A1 OR rs11200638 OR HtrA OR L56 OR PRSS11) and (macular degeneration OR wet-age related macular degeneration OR neovascular age-related macular degeneration OR AMD) and (VEGF OR vascular endothelial growth factor OR angiogenesis OR ranibizumab OR bevacizumab OR aflibercept). We also checked the reference lists of reviews and original articles by manually searching to check for additional studies not yet included in the above databases. All of the related articles had to be published before Mar 10, 2017.

Only published studies with full text articles were included in this meta-analysis. The inclusion criteria were as follows: (1) studies evaluating the relationship between rs11200638 and treatment response to wet-AMD; (2) independent prospective or retrospective association studies; and (3) studies with sufficient available data to estimate an odds ratio (OR) and its 95% confidence interval (CI). After abstracts were screened, the studies were read entirely to assess their appropriateness for the analysis. Meetings abstracts, case reports, editorial comments, review articles and letters were excluded.

### Data extraction and management

Two review authors selected the articles for inclusion independently. (Y.L.Z. and C.L.C.), based on the inclusion criteria. The following variables were extracted from each eligible study: surname of the first author, publication year, study type, ethnicity of the study population (Caucasian or East Asian), genotype distributions, mean age (years), frequency of risk allele A, duration of follow-up (months), treatment and study endpoints. Disagreements between two review authors were adjudicated by a third review author (Y.X.W.).

### Quality assessment

Two reviewers (Y.L.Z and C.L.C.) independently assessed the quality of all eligible studies using the Newcastle-Ottawa Quality Assessment Scale (NOS) [30]. A star system was used to judge the data quality based on three perspectives: patient selection, comparability of groups, and assessment of outcome. A nine-point scale of the NOS (range, 0–9 points) was used for the evaluation. Studies were considered to be of poor quality (scores of less than 4), medium quality (scores of 4–6) and high quality (scores of 7–9). Studies with NOS scores greater than 4 points were included in the final analysis. The quality of each study was awarded stars independently by the same two reviewers.

### Statistical analysis

We used the OR as the effective index, which was calculated for each study. To explore the possible associations between HTRA1 and anti-VEGF treatment for executive AMD, the following four genotype comparisons of ORs and their 95% CIs were calculated in the present meta-analysis: GG versus(vs) AA, GA vs AA, GG + GA vs AA, and allele G vs A. Further subgroup analysis was performed to examine the pharmacogenetic associations in Caucasian ethnic.

Heterogeneity assumptions were checked by the Q-statistic test and the *I* ^2^ statistic [31, 32]. If *P* < 0.10, heterogeneity was considered statistically significant for the Q-statistic test [32]*. I* ^2^ ranges from 0% to 100%. If the *I* ^2^ value of 0% indicates that heterogeneity does not exist, larger values indicate increasing heterogeneity. We chose fixed-effects models to pool the ORs when there was no heterogeneity among studies. Otherwise, the random-effects model was applied [31, 32]. A sensitivity analysis was performed by removing one study at a time to confirm that the stability of the meta-analysis results was not driven by any single study. Meta-regression was performed to detect the sources of heterogeneity [33]. Funnel plots and Egger’s test were used to detect publication bias (*P* < 0.05 indicated statistically significant publication bias) [34]. Notably, we did not have to check for departure from Hardy-Weinberg equilibrium (HWE) because all of the subjects in this meta-analysis were patients [35].

All the statistical analyses were pooled using Stata software (Version 12.0, Stata Corporation, College Station, Texas, USA). All of the tests were 2-tailed, and *P* < 0.05 was considered statistically significant.

## Results

### Literature search procedures and results

**Fig. 1:**
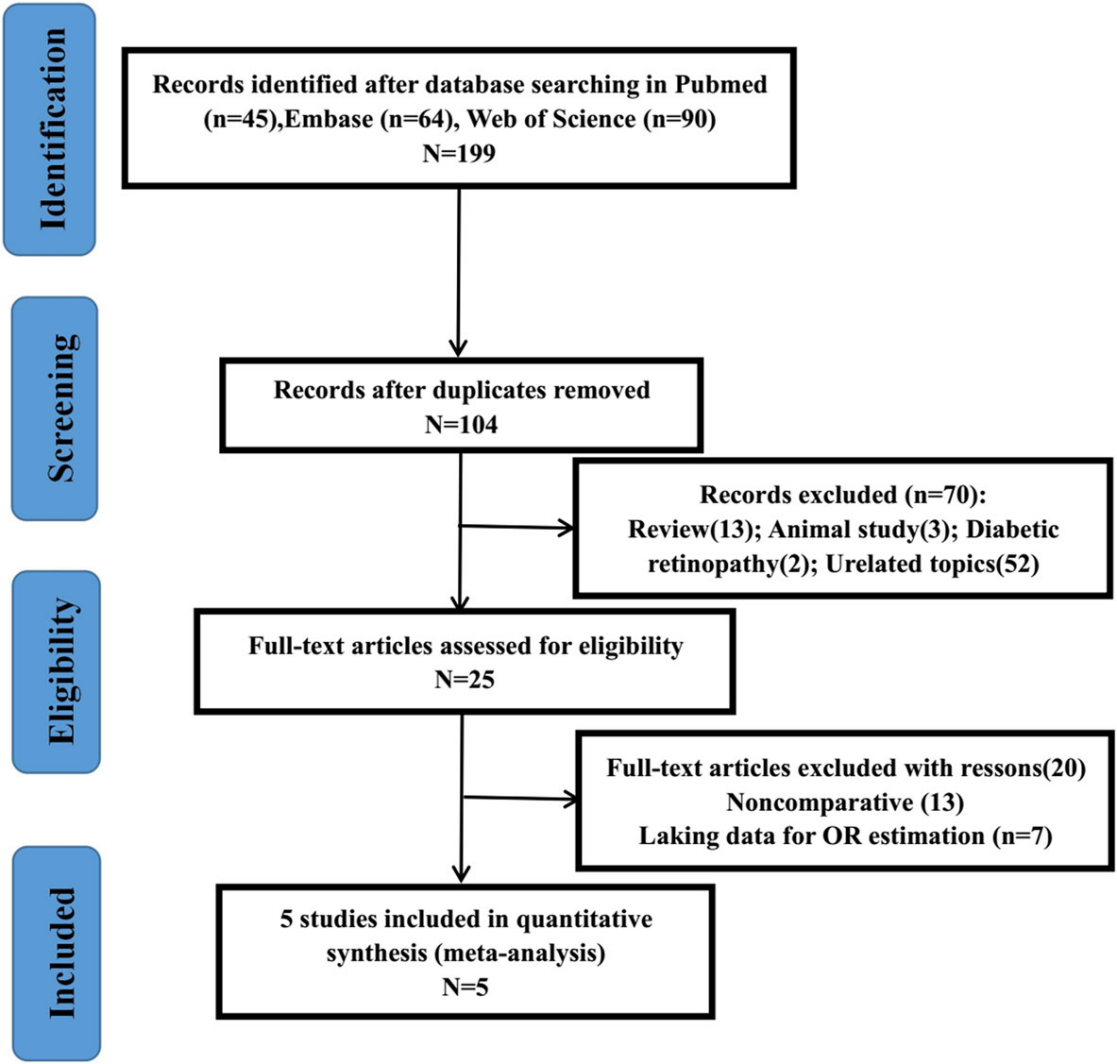
Flow chart of articles selection process

A flow diagram of the search procedure is provided in Fig. 1. The search strategy resulted in 199 relevant articles. After excluding 104 duplicates, 70 titles and abstracts were reviewed, and 25 studies investigated associations between the polymorphism rs11200638 genotypes and responses to treatment of exudative age -related macular degeneration were shown to be possibly relevant. Twenty of 25 studies failed to provide original data for OR evaluation or noncomparative studies were excluded. We had tried to contact with authors of original studies for data of odds ratios and continuous variable. Total 20 of them had no response) Five remaining studies were included in the meta-analysis [36–40], including 1570 cases, four of which with relatively small sample sizes, (*n* = 834) with a mean subject number of 184 (range, 104–273) [36–38, 40], except for the study by Hagstrom et al. [39].

The main characteristics of the eligible studies are listed in Table 1. Four studies were prospective, and one was retrospective. Regarding ethnicity, four of these studies were performed in Caucasians and one in East Asians. Two studies used ranibizumab, while 3 studies used either ranibizumab or bevacizumab. The duration of follow-up ranged from 5 to 24 months. We clarified the definition of positive/negative response. Some studies did not clearly define positive responders or negative responders, but this information could be obtained from the context.

**Table 1:** Main characteristics and quality scores of the included studies

Study (year)	Quality score	Study type	Ethnic	Number of cases	Mean age (years)	Treatment	Follow-up (months)	Definition of positive response	Study end points
McKibbin (2012) [36]	7	Prospective	Caucasian	104	81.5	RBZ	6	>5 BCVA letter score gain after 6 months	VA (EDTRS)
Orlin (2012) [37]	7	Retrospective	Caucasian	149	80.6	RBZ or BVZ	24	Improved/unchanged VA ≥ 24 months	VA (Snellen)
Abedi (2013) [38]	7	Prospective	Caucasian	210	78	RBZ or BVZ	12	<15 letters lose after 12 months	VA (EDTRS)
Hagstrom (2013) [37]	7	Prospective	Caucasian	834	78.5	RBZ or BVZ	12	≥15 letter gain from baseline	VA (EDTRS)
Park (2014) [40]	8	Prospective	East Asia	273	69.5	RBZ	5	≥8 letter gain at 5 months	VA (EDTRS)

*RBZ* ranibizumab, *BVZ* bevacizumab, *ETDRS* Early treatment diabetic retinopathy study, *VA* visual acuity

The genotype distributions of rs11200638 for all of the studies are summarized in Table 2. Four studies provided the number of positive responders or negative responders with GG/GA/AA genotypes; one by Abedi et al. focused on the comparison of genotype GG + GA vs AA. Moreover, the frequencies of the variant A allele of rs11200638 among all of the studies ranged from 40.86% to 68.61%, with the exception of the study by Abedi et al., which failed to assess allele and genotype distributions of GA and GG, respectively [38].

**Table 2:** Allele and genotype distribution of the rs11200638 polymorphism in studies included in the meta-analysis

	Positive Responders					Frequency (A) (%)	Negative Responders					Frequency (A) (%)
Study (year)	Genotype						Genotype					
	N	GG	GA	AA	G/A		N	GG	GA	AA	G/A	
McKibbin (2012) [36]	53	13	30	10	56/50	47.17%	51	14	25	12	53/49	48.04%
Orlin (2012) [37]	93	36	38	19	110/76	40.86%	56	21	24	11	64/46	41.07%
Abedi (2013) [38]	182	GG + AG = 148		34	NA	NA	28	GG + AG = 15		13	NA	NA
Hagstrom (2013) [39]	251	81	123	47	285/217	43.23%	583	193	275	115	661/505	43.31%
Park (2014) [40]	136	19	54	63	92/180	66.18%	137	17	52	68	86/188	68.61%

*NA* not avaliable

In terms of the predictive role of rs11200638 in treatment response with the genotype GG + GA vs AA, one of the five studies showed that the A allele tended to predict a poor response [38, 40], when performing genotype comparisons (GG vs AA, GA vs AA, G vs A) in all studies in which there were no statistically significant associations between response to anti-VEGF therapy and the genotype in both the positive-responder and negative-responder groups.

### Quantitative synthesis

**Fig. 2:**
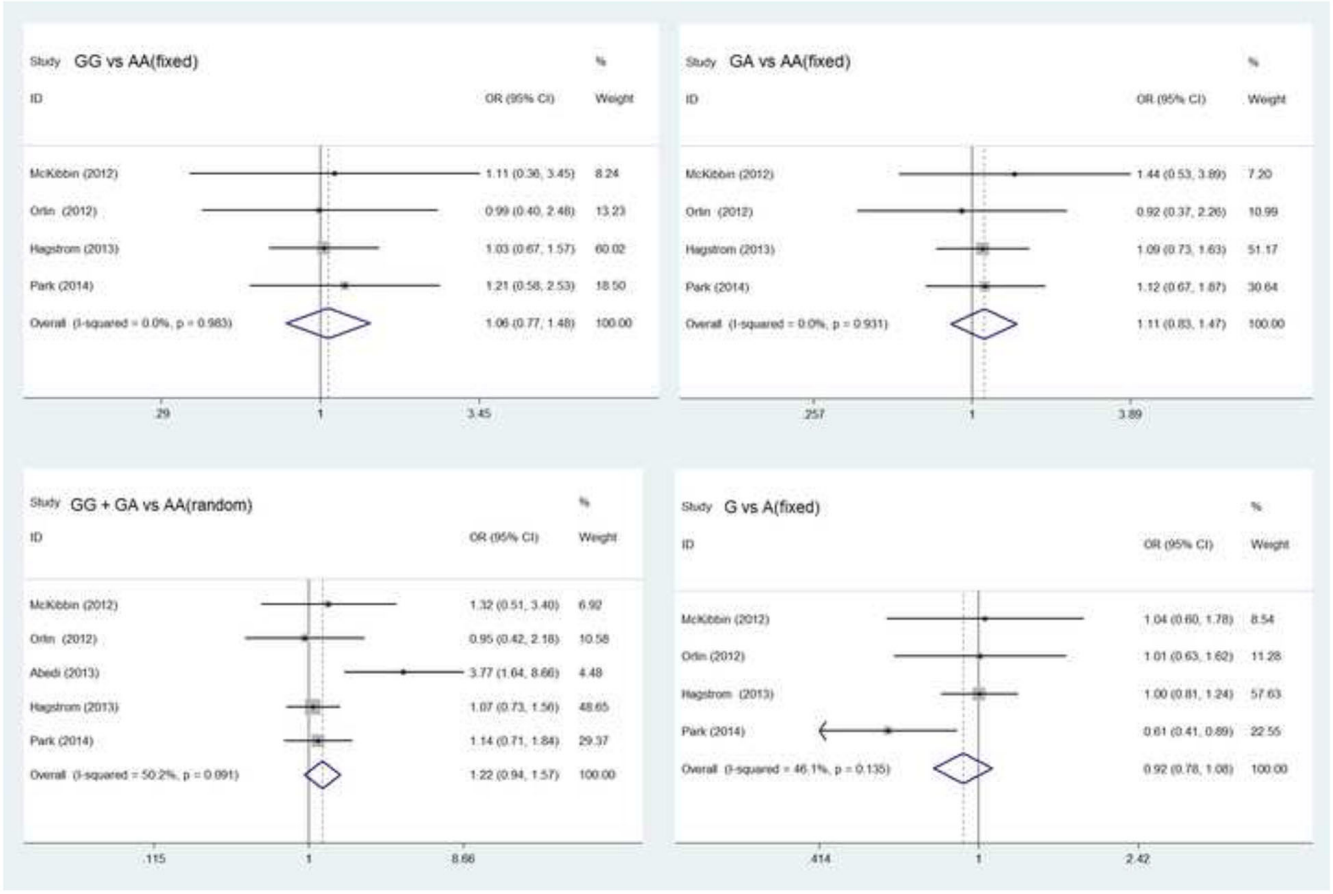
Forest plot of the association between genetic effects of rs11200638 polymorphism and anti-VEGF treatment of exudative AMD

**Fig. 3:**
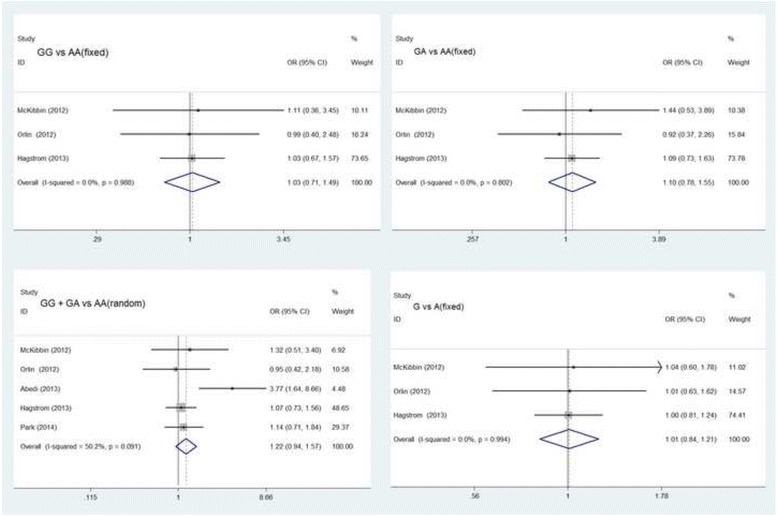
Subgroup analysis of the association in Caucasian ethnicity

We meta-analyzed the five included studies for the pooled associations between treatment response in neovascular AMD and rs11200638 genotypes. The results of all genotype comparisons (five studies for GG + GA vs AA, four studies for GG vs AA, GA vs AA, and G vs A) are shown in Table 3 (GG + GA vs AA: OR = 1.61 [95% CI 0.96 to 2.70], *P* = 0.07, random model; GG vs AA: OR = 1.16 [95% CI 0.84 to 1.60], *P* = 0.37, fixed model; GA vs AA: OR = 1.20 [95% CI 0.90 to 1.58], *P* = 0.21, fixed model; G vs A: OR = 1.04 [95% CI 0.69 to 1.56], *P* = 0.87, random model; Fig. 2). No significant differences were found in the above analysis. Further subgroup analysis was performed to examine the associations in Caucasian population, but these associations did not attain statistical significance in the analysis (Fig. 3).

**Table 3:** Subgroup and overall analysis for the association between genetic effects of rs11200638 polymorphism and anti-angiogenesis treatment of exudative AMD

						Heterogeneity	
Polymorphism		Subgroup	Study(n)	OR (95% CI)	*P* Value	*P* Value	*I* ^2^%
GG + GA vs AA	Ethnic	Caucasian	4	1.4 (0.81, 2.57)	0.22	0.05	62
	Overall		5	1.61 (0.96, 2.70)	0.07	<0.01	68
GG vs AA	Ethnic	Caucasian	3	1.03 (0.71, 1.49)	0.87	0.99	0
	Overall		4	1.16 (0.84, 1.60)	0.37	0.39	3
GA vs AA	Ethnic	Caucasian	3	1.10 (0.78, 1.55)	0.58	0.8	0
	Overall		4	1.20 (0.90, 1.58)	0.21	0.21	32
G vs A	Ethnic	Caucasian	3	1.01 (0.84, 1.20)	0.934	0.99	0
	Overall		4	1.04 (0.69, 1.56)	0.87	<0.01	75

*AMD* age-related macular degeneration, *OR* odds ratio, *CI* confidence interval

### Sensitivity analysis and meta-regression

**Fig. 4:**
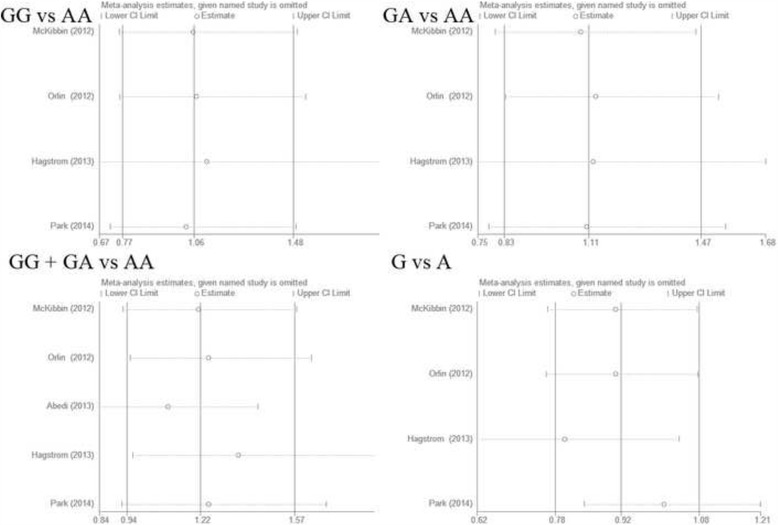
Results of sensitivity analysis in all genotype model

The summary ORs remained stable when removing one study at a time. We concluded that no study absolutely changed the relationship between rs11200638 and treatment response, indicating that the results of the present meta-analysis were relatively robust (Fig. 4).

**Fig. 5:**
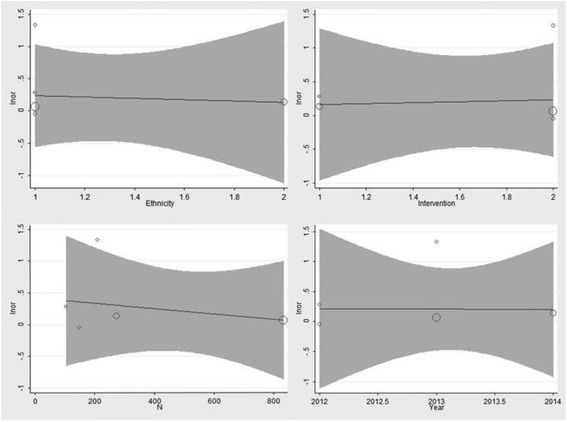
Results of meta-regression to analyze the heterogeneity of GG + GA vs AA genotype

We used meta regression to analyze the heterogeneity of GG + GA vs AA genotype. In view of the number of included literature was only five, we calculated single factor meta-regression, and the year, number of patience (N), ethnicity and intervention as independent variables (Fig. 5).

### Publication bias

Funnel plots and Egger’s test were performed to assess the publication bias of the included studies. In this meta-analysis, there were no obvious asymmetries of funnel plots in any genotypic comparison, while no statistically significant publication bias differences were found in the results of Egger’s test (GG + GA vs AA: *P* = 0.09; GG vs AA: *P* = 0.149; GA vs AA: *P* = 0.158; G vs A: *P* = 0.173).

## Discussion

Age-related macular degeneration is the leading cause of irreversible blindness in older individuals worldwide, especially the exudative type of AMD. Anti-VEGF treatment is currently the standard treatment for vision loss caused by exudative AMD [19–21]. Our study was based on a total of 4 cohort and 1 case-control studies involving 1570 cases to explore the associations between polymorphism rs11200638 in the HTRA1 gene and the response to treatment of exudative AMD specifically. However, both meta-analysis, focusing on the overall population, and the subgroup analysis by Caucasian ethnicity indicated that no statistically pharmacogenetic associations were found between the rs11200638 polymorphism and anti-VEGF treatment outcomes, with no evidence of publication bias. However, there has been strong evidence that the rs11200638 polymorphism can increase susceptibility to AMD, especially the neovascular type in Caucasian and Asian populations [12–16]. In addition, sensitivity analysis was conducted by removing one study at a time, and it showed similar and stable results, thus indicating that the results of the present meta-analysis were relatively robust. We used meta regression to analyze the heterogeneity of GG + GA vs AA genotype. We found that the several variables (the year, N, ethnicity and intervention) were not heterogeneous factors, the main research variables have little effect on the result of meta-regression. Additionally, the shapes of the funnel plots and the results of Egger’s test did not reveal obvious publication bias in this meta-analysis.

ARMS2 polymorphism rs10490924 (A69S) and HTRA1 polymorphism rs11200638 are usually show very similar genotypic distribution. Hu et al. reported significant association between A69S polymorphism rs10490924 and anti-angiogenesis treatment response [26], but present study found no significant pharmacogenetic association with HTRA1 rs11200638. This discrepancy may be caused by following reasons that there were 12 studies included in Hu et al., However, the number of included studies was much less than Hu et al., only 5 studies meet the inclusion criteria in present study. More studies are needed to further prove the conclusion of present study, especially well-designed and high quality prospective intervention studies. In addition, the clinical measures of response to anti-VEGF therapy not only including mean VA, but also the degree of anatomical response (fluid on OCT or FA, retinal thickness, change in total foveal thickness, change in lesion size). Both Hu et al. and this study were only focused on the VA change in pharmacogenetic association. It is not difficult to assume the ARMS2 polymorphism rs10490924 (A69S) and HTRA1 polymorphism rs11200638 may have consistence in other outcome indicators for pharmacogenetic association.

To minimize the bias of our research, we first searched in PubMed, Web of Science, and Embase with no limitations on language, and the studies had to be published in peer-reviewed journals; second, we collected data without ethnicity; and third, we assessed the quality of all of the studies using the NOS, and all of them showed high scores of 7–9. In order to improve the power of our study, we tried to ask original studies for data of odds ratios and continuous variable as well. Heterogeneity is a potential influence that might affect the results of the meta-analysis. However, no significant heterogeneity was observed in the overall population analysis of GG vs AA, GA vs AA, GG + GA vs AA, and G vs A, while in the subgroup analysis was based on ethnicity, heterogeneity was still not existed in the Caucasian population when performing comparisons for all genetic models.

Despite our rigorous methodology, some limitations of the current study should not be ignored. First, we cannot completely exclude publication bias by asymmetry plots because the number of included studies was insufficient. Second, the included studies were given adequate definition of positive/negative response. However, the criteria for positive/negative therapeutic response were various among studies although the measures of the response to treatment were just based on VA. In addition, treatment protocol, follow-up period, and study design is various. The underlying heterogeneity in treatment response can’t be completely ignored even though no significant heterogeneity was observed. Third, because of the complex nature of exudative AMD, many factors, including lifestyle, and other polymorphic susceptibility genes that also influence therapeutic outcomes, could not be completely excluded, so it is unsuitable to consider the HTRA1 gene definitely having nothing to do with exudative AMD treatment response. Fourth, the definition of response and the study endpoint were only changes in VA. Changes in central retinal thickness and maximum lesion thickness from baseline were also important treatment outcomes because anti-VEGF treatment could reduce the swelling of the central retina, which would decrease the central retinal thickness and be beneficial to VA improvement. However, studies included in this meta-analysis failed to find out any associations between these two important treatment outcomes and the genetic variants. Fifth, the number of studies included was limited. In addition, because some of the studies have insufficient available data to estimate ORs and its 95% CI [23–28], and two of them showed significant associations between the genetic variants of rs11200638 and patient response [27, 29], they were not included, and the conclusions must be validated by further studies. Finally, although all of studies included were of high quality, a limitation of these studies was that the non-randomized study design was used.

Despite these limitations, this study might be the first meta-analytic review evaluating the associations between polymorphism rs11200638 in the HTRA1 gene and the response to treatment of exudative AMD based on observational studies. No statistically significant pharmacogenetic associations were found between anti-VEGF treatment for exudative AMD and different genotypes in the HTRA1 gene in our meta-analyses. However, it seemed slightly early to draw a conclusion based on limited numbers of studies available so far, especially only studies providing sufficient available data to estimate values of ORs. Future research, especially well-designed and high quality randomized, controlled trials or interventional studies, are highly desirable to enable more precise estimates and better understanding of the relationship between polymorphism rs11200638 in the HTRA1 gene and responses to anti-angiogenesis treatment for exudative AMD.

## Conclusions

In conclusion, our meta-analysis indicated that there was no association between the polymorphism rs11200638 in HTRA1 gene and response to anti-VEGF treatment of exudative AMD. However, more studies are needed to further prove the conclusion of present study, especially well-designed and high quality randomised controlled trials or intervention studies.

## Abbreviations

AMD: Age related macular degeneration;
ARMS2: Age-related maculopathy susceptibility 2
CFH: Complement factor H
CIs: Confidence intervals
CNV: Choroidal neovascularization
HTRA1: High temperature requirement factor A1
HWEL: Hardy-Weinberg equilibrium
NOS: Newcastle-Ottawa Scale
ORs: Odds ratios
PRISMA: Preferred Reporting Items for Systematic Reviews and Meta-Analyses
RPE: Retinal pigment epithelium
SNPs: Single nucleotide polymorphisms
VA: Visual acuity
VEGF: Vascular endothelial growth factor
VEGFA: Vascular endothelial growth factor A
VEGFR2: Vascular endothelial growth factor receptor 2
vs: Versus
